# Parents' assessment of parent-child interaction interventions – a longitudinal study in 101 families

**DOI:** 10.1186/1753-2000-3-8

**Published:** 2009-03-10

**Authors:** Kerstin Neander, Ingemar Engström

**Affiliations:** 1 School of Health and Medical Sciences, Psychiatric Research Centre, Örebro University, Örebro, Sweden

## Abstract

**Background:**

The aim of the study was to describe families with small children who participated in parent-child interaction interventions at four centres in Sweden, and to examine long term and short term changes regarding the parents' experience of parental stress, parental attachment patterns, the parents' mental health and life satisfaction, the parents' social support and the children's problems.

**Methods:**

In this longitudinal study a consecutive sample of 101 families (94 mothers and 54 fathers) with 118 children (median age 3 years) was assessed, using self-reports, at the outset of the treatment (T1), six months later (T2) and 18 months after the beginning of treatment (T3). Analysis of the observed differences was carried out using Wilcoxon's Signed-Rank test and Cohen's d.

**Results:**

The results from commencement of treatment showed that the parents had considerable problems in all areas examined. At the outset of treatment (T1) the mothers showed a higher level of problem load than the fathers on almost all scales. In the families where the children's problems have also been measured (children from the age of four) it appeared that they had problems of a nature and degree otherwise found in psychiatric populations. We found a clear general trend towards a positive development from T1 to T2 and this development was also reinforced from T2 to T3. Aggression in the child was one of the most common causes for contact. There were few undesired or unplanned interruptions of the treatment, and the attrition from the study was low.

**Conclusion:**

This study has shown that it is possible to reach mothers as well as fathers with parenting problems and to create an intervention program with very low dropout levels – which is of special importance for families with small children displaying aggressive behaviour. The parents taking part in this study showed clear improvement trends after six months and this development was reinforced a year later. This study suggests the necessity of clinical development and future research concerning the role of fathers in parent-child interaction interventions.

## Background

### Parent-child intervention

Finding ways to prevent mental health problems is perceived as an important task within child psychiatry, in concurrence with other authorities and organizations striving to promote the course of children's development. Since the 1960s the arena of early childhood interventions has been transformed from a modest collection of pilot projects to a multidimensional domain of theory, research, practice and policy [1]. Such interventions were previously directed towards the children themselves – specifically targeting the needs of disabled children and children growing up in poverty [2]. The scope and the target group for these interventions have since then broadened and may now include mental health problems at large. As research in the field of child development has grown, the proliferation of parent-child and family interventions have reflected our increased understanding of the critical and determinative nature of parent-child interaction [2]. Early childhood intervention has thus experienced a paradigm shift from a child-oriented to a family-oriented approach [3].

The main theoretical basis generally applied for this type of intervention is attachment theory [4,5] which emphasizes the importance of the quality of early relationships [2]. A core feature of this theory is the importance for a child to experience everyday interaction with a reasonably sensitive and sufficiently predictable parent able to provide a "secure base" [6] from which the child can comfortably engage with the world, balancing inquisitiveness with a need for security.

This theory is often complemented by the ecological perspective [7], which highlights both the interaction of the child as a biological organism within its immediate social environment in terms of processes, events and relationships and the interaction of social systems in the child's social environment [8]. Within the transactional model [9] the development of the child is seen as a product of continuous dynamic interactions between the child and his or her family and social context. In this web of transactional processes, of which the child and his/her parents form part, researchers have been able to empirically identify a number of aspects that have proved to be important for a positive development of the child; parental stress [10], parental patterns of attachment [11,12], parents' mental health and well being [13], parents' access to a social network [14], and the possibilities of obtaining social support [15].

Among the seminal contributions to the fields of infant development and parent-child treatment, the writings of Daniel Stern [16-18] have offered critical and highly influential new theoretical perspectives. Stern describes the clinical system shaped during parent-child interventions and emphasizes that the interaction includes the inner representations of the child and the parent as well as their observable behaviour. These aspects constantly influence each other and the intervention can therefore choose different *ports of entry *to achieve change – for example the parent's inner images of the child, the representations of himself/herself as a parent, or the observable interaction. Stern [19] stresses the fact that the therapeutic alliance in parent-child treatment must be far more positive and validating than in a traditional psychodynamic therapeutic context.

### Studies on the efficacy of interventions

The first systematic survey of interventions specifically directed towards the parent-child interaction, based upon attachment theory, was undertaken by van Ijzendorn et al [20]. This survey, including twelve mother-child interventions, supported the theory that such interventions increased the mothers' sensitivity, but the effect on the children's attachment was surprisingly weak. This result indicated the influence of parental attachment representation on children's attachment through mechanisms other than responsiveness; referred to as "the transmission gap" [21]. A narrative review by Egeland et al [22] of 15 attachment-based interventions pointed out that there are many factors at different ecological levels that may interfere with successful intervention. The source of obstacles to a secure parent-child attachment may be found in the child, the caregiver, the care-giving environment, or a combination of all these. In order to meet the participants' needs, the authors recommend flexible broad-based interventions – particularly for high-risk samples, where the parents are often dealing with multiple challenges and barriers in their own lives. Such comprehensive interventions should be designed to make services available that can meet both the attachment-related and other needs of high risk families; e.g. enhancing parental well being and providing and promoting social support.

A different conclusion was reached by Bakermans-Kranenburg et al [23] in a meta-analysis of interventions with the purpose of enhancing parental sensitivity and/or child attachment security. This review comprises 70 studies where the intervention started at an average child age of below 54 months. The intervention studies were not restricted to a specific population: both middle-class samples with healthy children, at risk populations, and clinical samples were included. The analysis revealed that the interventions had an impact both on the mothers' sensitivity and – to a lesser degree – on the children's attachment. Interventions with video feedback were found to be more effective than those without. The most effective interventions used a moderate number of sessions and focused on sensitivity in families with, as well as without, multiple problems. These findings were summarized in the title of the article: Less Is More. Only three of the studies included fathers and these studies are all fairly old [24-26] but the conclusion in the review was that interventions including fathers appeared to be significantly more effective than interventions focusing on mothers only.

It has thus been shown that early interventions directed towards parent-child interaction may have a positive effect upon parenting [23], but whether "less is more" or "more is better" is an issue that can only be resolved through further studies [27].

A critical analysis of interventions based on attachment theory, limited to research that has been peer-reviewed, paid special attention to methodological aspects of the primary studies [28]. The conclusions, based upon 15 prevention studies published between 1988 and 2005, revealed that attachment interventions produce on average weak to moderate effects across caregiver and child outcomes. In only one of the studies were fathers involved. The authors emphasize that data on treatment integrity or social validity – if the interventions are accepted by key agents e.g. parents, children and intervention agents – are essentially nonexistent in the literature. This is significant since an intervention must be accepted by important participants in order to have high effectiveness under real-world conditions – and not only high efficacy under tightly controlled research conditions. Naturalistic studies, i.e. studies carried out under real-world conditions have a special value in so far as they can provide answers concerning treatment acceptability by giving information about dropout from treatment, which may be seen as a proxy for acceptance of treatment. Egeland et al [22] ask for more research on interventions based upon the ecological model taking into account such factors as social support and parents' emotional health and well-being. Bakermans-Kranenburg et al [23] stress the need for long-term follow-up studies, since sleeper effects – effects that emerge a long time after the intervention – on for example attachment security might otherwise remain undetected.

### Cultural considerations

It is also of great importance to study parent-child interventions within various cultural contexts. Even though the development of such interventions has been considerable for the last thirty years in Sweden as well, only a small number of these have been assessed with regard to outcome [29,30]. There are cultural variations with regard to children's mental health. Heiervang et al [31] have shown that the Norwegian prevalence of externalising disorders (behavioural and hyperactivity) was about half that found in Britain, whereas rates of emotional disorders were similar. Differences like this offer a rationale for the study of parent-child interventions in different cultural contexts. Research results from the Nordic countries – with their resources in the field of mother and child health care, parental leave, and a well-developed pre-school – may be of specific interest to complement and enhance knowledge about various conditions for these interventions. The most obvious deficit in this research field hitherto is, however, the almost complete lack of intervention studies that include fathers.

### A Swedish example of parent-infant intervention approaches

This study is based on an intervention programme that has been developed during the last two decades in Sweden. Attachment theory [4,5] along with an ecological, transactional perspective [7,9] and Stern's theories of development in infancy [16] and of preconditions for treatment [17,18] provide the theoretical foundation employed at these centres. Attachment theory, which is usually associated with infants and small children, is also relevant for families with children in their middle childhood (7–12), when attachment to the parent(s) is still salient and important [32] though with a somewhat altered goal: from proximity of the attachment figure in early childhood to his/her availability in middle childhood according to Bowlby [33]. This gradual development is taken into consideration in the therapeutic work. A salutogenetic [34] therapeutic approach implies a focus on factors that support a positive development and not only an interest in factors that cause problems.

#### The work assignment

The linchpin of the therapeutic work is the *collaborative relationship *between the parent(s) and the therapist. A basic principle is that *the goals of intervention *should be established through a dialogue between the parents and the therapist based on the parents' own descriptions of the problem with the changes they desire being crucial. Priority is given to the parents' interpretation of the problem. This means that even though both the person referring the family and the therapist may suggest themes to work with, it is always the parents who decide what problem areas are ultimately selected as the focus of the treatment, as long as this is in accordance with the therapist's competence and role. The interventions may concentrate on outer, observable behaviour and/or on the inner images the parent has of his or her child and him or herself. The dialogue leads to the agreement upon a work assignment, which also entails clarification of the roles of the practitioners and the parents. On the basis of these discussions the professionals endeavour to shape the treatment according to the pronounced needs of each family.

#### Elements in the program

The intervention comprises a number of elements combined on the basis of the needs of the family in conformity with the ideas behind stepped care, which refers to the practice of beginning therapeutic measures with the least extensive intervention possible and moving on to more extensive interventions only if deemed necessary in order to achieve a desired therapeutic goal [35]. The first step – which is *always *involved but which *never *constitutes the entire intervention – is parental counselling. The next step – which comprises the main element of the intervention – is *interaction treatment *which can be carried out in different forms as described below; "in video", "in vivo" (live), and "in verbis" (verbally). A combination of these three forms is most often used. When required, collaboration with the family's social network forms yet another step.

#### Interaction treatment "in video" – Marte Meo

Marte Meo was developed in the Netherlands by Maria Aarts in the 1980s [36], and may be regarded as an application of modern developmental psychology [16]. The starting point in the Marte Meo intervention is the question raised by the parent. The therapist makes a short video recording (3–7 minutes) of the child interacting with his/her parent(s) and analyses it, using a number of basic principles for a natural supportive dialogue. The principles the therapist is looking for are whether and how (1) the child's focus of attention is recognized by the parent, (2) the child's states, initiatives and feelings are acknowledged by the parent, (3) the child is given the time and space to react, (4) the child's ongoing actions, experiences and feelings are interpreted, punctuated and named by the parent, (5) the child is assisted to experience structure and predictability, (6) the child is guided by well-adjusted information and gets approving confirmation when a desirable behaviour is emerging, (7) the child is assisted through inevitable unpleasantness, (8) the child is encouraged to take an interest in other persons and their actions and feelings/sentiments, and (9) the child is helped to start and close an activity or a dialogue [37]. The therapist then chooses sequences to review with the parent, to create a link between the parent's initial question and the therapist's idea of what kind of support the child needs. The basic purpose is to afford an opportunity for joint observation and reflection on the child and his/her needs. The sequences selected are preferably ones that contain "moments of solutions" where the child is provided with the support he/she needs and the parent thus becomes his/her own model. The second best choice is where the needs of the child are displayed. The parent becomes an active, reflective participant in the work of developing his/her interaction with the child, and the child is mentalized instead of problemized [37]. The parent is encouraged to practise in everyday situations, and the process continues with new recordings, analyses and joint reflections.

#### Interaction treatment "in vivo"

Modern developmental psychology and attachment theory emphasize the quality of the everyday interaction for the development of the child. In interaction treatment "in vivo" the therapist and the parent use ordinary everyday life situations as points of departure. The work is framed by the work assignment and the situations can be planned by the therapist and the parent(s) together or utilized as they arise. Interaction treatment "in vivo" always includes the child and can take place in the homes of the families or/and at the centres, in a group setting or with only one family and the therapist partaking.

Interaction treatment "in vivo" is guided by the same understanding of a child's need for dialogue as Marte Meo. Since the structure is less well-defined "in vivo", the therapist faces other challenges, e.g. not to make up for the support the child needs but is not given by his/her parent. The parent is encouraged to become more attentive to the focus of attention of the child, his/her initiation of dialogue, expressions of emotions, rhythm and the child's need of assertion, guidance and protection. The aim of this part of the treatment is to enhance the parent's own ability to mentalize [38], i.e. to imagine how the world is conceived from the child's perspective, which may be of crucial significance in parenthood. Moments of intersubjectivity – the sharing of lived experience – are considered indispensable both for the therapeutic relationship and for the child's development [18].

In accordance with attachment theory, special attention is given to those factors which, alongside sensitive attunement, are thought to be of the greatest importance in helping the child to experience that his/her parent is providing a secure base and a safe haven. This must be communicated to the child through the parent's behaviour and includes for instance that the parent is not perceived as frightened/frightening, that he/she is not explicitly hostile, that the parent shows a fundamental willingness to soothe and comfort in times of fear and distress [39] and that he/she is predictable in his/her reactions and actions.

Interaction treatment "in vivo" involves the joint reflection of therapist and parent and the child may also take part if that is felt to be appropriate with regard to age and other circumstances.

#### Interaction treatment "in verbis" (verbally)

The port of entry in interaction treatment "in verbis" is the parent's representations, e.g. his/her inner pictures of the child or of himself/herself as a parent. There may also be focus on the parent's own attachment history. It might for example be of help for parents to reflect upon how their own avoidant attachment behaviour was quite an appropriate strategy when they were children, but that the situation is now different, with new possibilities both in relation to their partners and in their ways of meeting their own children's needs of a secure base. Parents may also have a strong wish not to repeat their own parents' way of bringing up children – for example by using threats or violence – but realize that they lack alternative models.

Obstacles in the parent's history are often referred to as "the ghosts in the nursery" [40], but together with the exploration of painful memories it can be valuable to identify "the angels in the nursery", i.e. the beneficial experiences [41].

#### Collaboration with the families' social network

In accordance with the ecological perspective, collaboration with the families' private and professional network is also often taken into account. The aim may be to give the family access to resources from other micro-systems; to develop connections fraught with conflict between micro-systems (e.g. the family and the child-care); or to coordinate multiple micro-systems involved in network meetings.

### Aims of the current study

This longitudinal multi-centre study includes fathers, mothers and children in parent-child interaction interventions at four treatment centres in Sweden. Since one of the fundamental principles behind these interventions is that the parents have the right to define the problems and to take an active part in planning the intervention, it is logical to focus on the parents' experience of change. The self-report measurements used in this study cover those areas, presented earlier in the text, that have been shown to be of importance for good parenting and child development.

The aim of this study was

• to describe families – where difficulties in the interaction between parents and children have led to participation in parent-child interaction interventions at four centres in Sweden – with respect to social characteristics and psychological aspects of scientifically proven importance. These aspects were: the parents' experience of parental stress, parental attachment patterns, the parents' mental health and life satisfaction, the parents' social support and the children's problems at the outset of the treatment (**T1**)

• to examine long term changes (18 months after beginning of treatment (**T3**)) and short term changes (6 months after beginning of treatment (**T2**)) regarding the same aspects as those assessed at the outset of the treatment.

### Ethical approval

This study has been approved by the Research Ethics Committee of Orebro # 319/02.

## Methods

### The four centres for parent-child intervention

The families included in this study have participated in treatment at one of the following four centres for parent-child intervention in Sweden: Gryningen in Karlskoga (ages 0 – 6), Lindan in Lindesberg (ages 0 – 5), Lundvivegården in Skövde (ages 0 – 12) and Björkdungen's family centre in Örebro (ages 0 – 12). Gryningen is run by the Department of Child and Adolescent Psychiatry in collaboration with the Social Welfare authorities, Lindan by the Department of Child and Adolescent Psychiatry while Lundvivegården and Björkdungen fall under the auspices of the Social Welfare authorities. They are all outpatient departments. Treatment is voluntary, but some parents may nevertheless feel themselves coerced into complying with the wishes of social authorities for them to participate in the intervention.

The therapists at the centres all have degrees (e.g. social workers, preschool teachers) and have been trained in the Marte Meo method. Some of the therapists have acquired additional qualifications in, for instance, cognitive psychotherapy and family therapy.

In spite of organizational differences at the centres, the shared theoretical foundation, essential features in their therapeutic approach and the elements in the intervention programme (described above) justify the idea of including them all in a multi-centre study.

### Subjects

**Figure 1:**
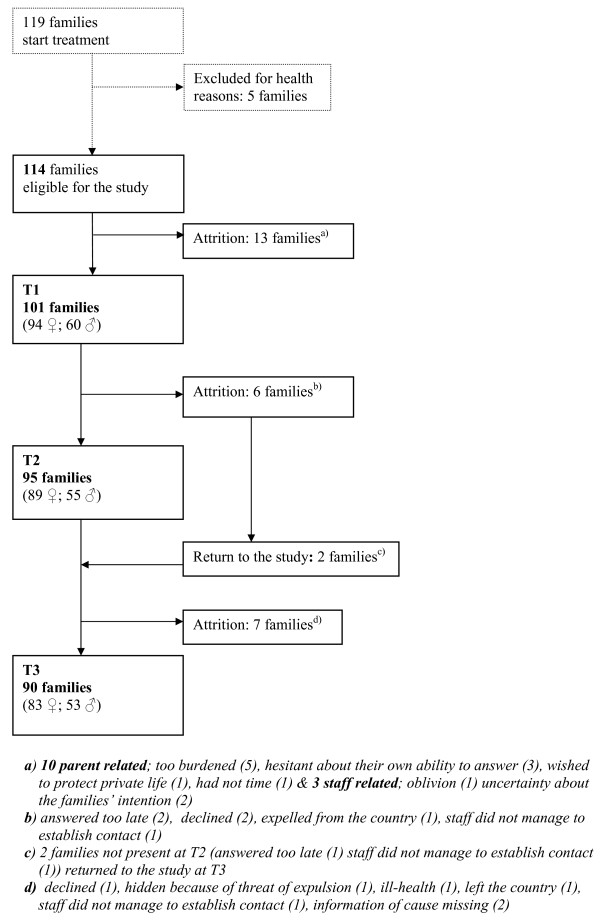
Study flowchart.

This study is based on a consecutive sample of all parents who commenced treatment during three years at one of these four centres (Figure 1). The study excluded parents displaying substantially impaired cognitive capacity due to acute and serious mental reactions. Of the five families excluded for that reason, four were refugees seeking political asylum. In all, 154 parents (94 mothers and 60 fathers) in 101 families agreed to participate in the study. In the 54 two-parent families all of the mothers and 45 (83%) of the fathers participated in treatment.

Altogether the 101 families had 118 children taking part in the treatment (Table 1). Forty-four (37%) of these were girls and 74 (63%) were boys. The children's ages varied from unborn (the treatment started towards the end of pregnancy) up to 12-year-olds, with a median age of 3. The parents' ages varied between 18 and 49 with a median age of 31.

**Table 1:** Subjects & contact initiators

			**n**
**Children's age (n = 118 children; 44 girls & 74 boys)**	♀	♂	
Unborn	0	4	4
0 – 11 months	10	6	16
1 year	1	6	7
2 years	7	11	18
3 years	6	10	16
4 years	4	10	14
5 years	4	7	11
6 years	1	6	7
7 years	2	4	6
8 years	1	5	6
9 years	2	1	3
10 years	4	1	5
11 years	0	3	3
12 years	2	0	2
			
**Child's residence (n = 101 families)**			
Mother & Father			54
Single Mother			26
Mother & Stepfather			9
Alternating residence (at least 10 days a month with each parent)			6
Single Father			4
Father & Stepmother			1
Foster home			1
			
**Parents' occupation (n = 154 parents, 94 **♀**; 60 **♂**)**	♀	♂	
Employed	35	41	76
Unemployed/employment measures	15	11	26
Long-term sick-leave/temporary disability pension/pension	24	2	26
Student	13	3	16
Seeking political asylum	3	3	6
Working in the home	3	0	3
Information missing	1	0	1
			
**Initiating contact **(≥ 1 per case; 124 contact initiators in 101 families)			
Social services			48
Parents			37
Adult psychiatry			12
Child health service			11
Paediatric clinic			4
Preschool			3
Child psychiatry			3
Maternity welfare			2
Other			4

Of the 154 parents (94 ♀; 60 ♂) in the study 131 (77 ♀; 54 ♂) were born in Sweden. There were 10 foreign-born parents (7 ♀; 3 ♂) from European countries and 11 parents (10 ♀; 1 ♂) from countries outside Europe (data is lacking for two of the fathers). This means that Swedish-born parents were somewhat overrepresented in the study compared to society as a whole, but the parents born abroad dominated among the parents excluded for reasons of health. One-third of the parents taking part in the study were either unemployed or on sick leave, which constitutes a considerably higher proportion than in the population as a whole.

### Contact initiators and contact causes

The parents may themselves contact the centres or be referred to them by child health care, social services, preschools or some other body (Table 1). Contact cause (Table 2) is always related to the interaction between the parent and the child. When, for example, a parent's poor self-esteem is indicated as the cause of contact, it is therefore its impact on the parent-child relationship that is the reason for contact. Contact causes shown in table 2 refer to what was indicated when the parents applied to the centres or were referred to them. It is not, therefore, an assessment made by the staff at the centres. Dysfunction in parent-child interaction was the most common reason for seeking treatment. The predominant cause with reference to the children was externalizing behaviour and it is worth noting that aggression was by far the most frequent cause for contact. These are examples of how the parents expressed their goals for the treatment: "to put an end to Oscar's biting and fighting", "to feel confident as a mother of my baby", "to help Anna to concentrate on one thing" or "to be able to communicate with Alan without constant trouble".

**Table 2:** Contact cause (≥ 1 per case)

	**n**
**Interaction between parent/parents – child (174 causes stated in 91 families)**	
Need for support in the parent role	58
Interaction difficulties	53
Boundary setting problems	44
Attachment difficulties	14
Suspected abuse	4
Other	1
**Child (142 causes in 78 children in 75 families)**	
Externalizing problems	81
*Aggressiveness (37), Hyperactivity & concentration problems (31)*,	
*Cannot/Does not want to listen/obey (7)*,	
*Troublemaking/Obstinacy/Acting out (6)*	
Regulation problems	31
*Sleeping (17), Feeding (7), Screaming (5), Toilet training (2)*	
Contact difficulties	5
Interaction difficulties with siblings and/or other children	5
Internalizing problems	4
Delayed development	6
Handicap/illness	6
Trauma	3
Other	1
**Parents (89 causes in 70 parents in 55 families)**	
Mental problems/mental illness	35
Insecurity/low self-esteem/immaturity/very young	38
Worn-out & tired	6
Abuse	4
Feeling of loneliness	2
Somatic illness	2
Assaulted others	1
Other	1
**Relationship between the parents/step-parents (47 causes in 35 families)**	
Conflict or crisis with the partner/the other parent	18
Separation	17
Violence or threat of violence	3
Death	3
Other	6
**Social network (32 causes in 27 families)**	
Insufficient network	12
Conflict filled network	18
Other	2
**Social situation (25 causes in 23 families)**	
Burdened social situation	17
Strains in connection with refugee situation	5
Other	3

### Treatment, duration, compliance, and termination

The interaction treatment consisted of various combinations of the three modalities "in video", "in vivo" and "in verbis" (Table 3). Collaboration with the families' social network was reported for 60% of the families, most frequently with child-care and school followed by social services and relatives.

**Table 3:** Interventions in 101 families

	**Families**	**Number of sessions**		
	n	Md	Mean	Sd
**Interaction treatment**	**101**			
**"In vivo"**	**88**	21.5	32.3	28.5
*At a centre – family & therapist in a group setting*	*56*	*33.5*	*39.1*	*28.9*
*At a centre – family & therapist exclusively*	*22*	*9.0*	*12.8*	*12.4*
*At home*	*57*	*4.0*	*6.9*	*7.9*
**"In video" Marte Meo**	**83**	6.0	6.1	3.6
*Reviews with one parent*	*67*	*4.0*	*4.7*	*3.2*
*Reviews with two parents*	*44*	*4.0*	*4.5*	*2.7*
**"In verbis"**	**95**	9.0	13.0	12.3
*Number of sessions with one parent*	*74*	*6.5*	*8.9*	*8.5*
*Number of sessions with two parents*	*60*	*6.0*	*9.3*	*8.7*
				
**Combinations of treatment modalities**				
"In vivo" & "In video" & "In verbis"	72			
"In vivo" & "In verbis"	13			
"In video" & "In verbis"	5			
"In vivo" & "In video"	3			
"In verbis"	5			
"In video"	3			
"In vivo"	0			
				
**Collaboration with the families' social network**	**61**			
Child care (24) & School (8)	32			
Social services	31			
Relatives	24			
Psychiatry (adults)	8			
Child health care	7			
Child psychiatry	6			
Friends	3			
Maternal health care	1			
Network meeting	9			

If a family or a member of a family was receiving services at the outset of treatment from e.g. a psychiatric outpatient unit, these services generally continued during the intervention time since the centres have no wish to act as a substitute for other agencies.

After six months (T2) 74 of the 101 families were still in treatment, and when the final assessment (T3) took place – 18 months after the outset – treatment was still under way for 19 families (Table 4). For the families that had completed treatment at T2 or T3, the time of treatment varied from 1 to 18 months. The median treatment period for all 101 families was 10 months. Slightly more than a third of the families attended treatment once a week, half of them more often (maximum three days a week) and the rest less frequently. Failure to attend treatment was low for almost three-quarters of the families (≤ 15% of planned treatment sessions).

**Table 4:** Treatment duration, compliance and termination

	**Families**	**Number of months**		
	n	Md	Mean	Sd
**Treatment duration**				
Length of treatment for all 101 families	101	10		
*Treatment completed*	*72*	*8*	*8.9*	*4.60*
*Interrupted treatment*	*10*	*8*	*9.5*	*6.12*
*Still in treatment at T3*	*19*			
				
**Treatment completion at T2 (6 m) & T3 (18 m)**				
Treatment completed at T2	24			
Treatment interrupted at T2	4			
Treatment completed at T3 *(another 48 families)*	72			
Treatment interrupted at T3 *(another 6 families)*	10			
				
**Proportion of failure to attend treatment (101 families)**				
≤ 15% of planned treatment sessions	73			
16 – 25%	9			
26 – 50%	14			
51 – 75%	1			
≥ 76%	2			
Missing data	2			
		**After n months**		
**Treatment interruption**	**10**			
Families moved from the neighbourhood	3	*6; 7; 19 months*		
Asylum seeking families expelled from the country	2	*1; 11 months*		
Investigation by social services	2	*4; 9 months*		
Staff reasons: sick leave or end of service	3	*5; 15; 18 months*		

Out of the 101 families taking part in the study, treatment was interrupted for a total of ten families: three families moved from the neighbourhood, two families seeking political asylum were expelled from the country, two families were subject to child welfare assessments by the social services and finally there were three families whose treatment was interrupted because of staff reasons: sick leave or retirement. The median length of treatment for these ten families was eight months. There were no other dropouts from the treatment.

### Measures

#### The parents' experience of parental stress

The Swedish Parenthood Stress Questionnaire (SPSQ) [42] is based on the Parent Domain of the Parenting Stress Index [43]. This instrument comprises of five subscales: incompetence, role restriction, social isolation, spouse relationship problems, and health problems. The total experience of stress is measured by a general parenting stress scale consisting of all items. The instrument has been used in several studies and has displayed good psychometric properties [42]. Since about half of the families seeking help at the four centres are single parents a special "single version" was designed for them in which the questions regarding the sub-scale on spouse relationship problems had been removed.

#### The parents' patterns of attachment

The Relationship Questionnaire (RQ) [44] is a self-report instrument designed to measure four categories of attachment (avoidant/dismissive; secure/autonomous; ambivalent/preoccupied and disorganized/fearful), using combinations of a person's self-image (positive or negative) and image of others (positive or negative). On the RQ the respondent is asked to rate, on 7-point scales, how well he/she feels the description of the four patterns apply to their own experiences. The psychometric properties of the Swedish version have proved to be satisfactory [45].

#### The parents' mental health

The instrument used to measure psychological health was the General Health Questionnaire 12 (GHQ12) [46], a questionnaire with 12 questions. The index can vary between the values 0 and 12, with a low value indicating good psychological health. The threshold value for poor psychological health is 3 [47]. The instrument has displayed good psychometric properties [46].

#### The parents' present and expected life satisfaction

Cantril's Self-Anchoring Ladder of Life Satisfaction [48] is a measure of an individual's overall assessment of life satisfaction. Subjects are asked to evaluate their life at the present time, one year ago and one year from now on a ladder, with the bottom (0) representing the worst possible life and the top (10) the best possible life. The Cantril Ladder has been reported to have good validity and stability and reasonable reliability [49].

#### The parents' social support

In order to obtain a measure of perceived availability and adequacy of support from intimates and the wider social network we used a brief version of The Interview Schedule for Social Interaction [50]. The Swedish version [51] consists of 30 items measuring both the availability and the adequacy of attachment and social interaction and is divided into four subscales. The maximum obtainable scores are: for Availability of Social Integration (AVSI) 6 points, Adequacy of Social Integration (ADSI) 8 points, Availability of Attachment (AVAT) 6 points, and Adequacy of Attachment (ADAT) 10 points, 1 for each item. The ISSI has displayed good psychometric properties [52].

#### The children's strengths and difficulties

The Strengths and Difficulties Questionnaire (SDQ) [53] is a brief behavioural screening questionnaire concerning 3–16 year olds. It exists in several versions: the versions used in this study were questionnaires for completion by the parents of 4–16 year olds. In this study there were 50 families with children 4 years or older. All versions of the SDQ incorporate statements regarding 25 attributes, some positive and others negative. These 25 items are divided into 5 sub-scales: emotional symptoms; conduct problems; hyperactivity/inattention; peer relationship problems and prosocial behaviour. The first four sub-scales produce a total difficulties score. The SDQ also includes an impact supplement. The instrument has been translated into Swedish and its psychometric properties are considered good [54,55].

### Procedure

The first point of assessment called **T1 **took place at the outset of the treatment, the second assessment (**T2**) six months later and the third point (**T3**) 18 months after treatment began. In order to minimize attrition, members of the staff contacted the families and asked them to come to the centres to fill in the questionnaires if they were no longer undergoing treatment at T2 and T3. If this was not possible, the questionnaires were sent home to the family. There was no loss of data from the great majority of informants. The exact number of persons completing each questionnaire is indicated in tables 5, 6 and 7. The staff at the four centres supplied information for the *Background data *(at T1) and a *Treatment Journal *(at T2 & T3) with data concerning the intervention.

### Statistical analysis

The results of the assessments made by the parents at the outset of treatment (T1) were compared with available community and clinical samples. No individual data were accessible from these studies, which ruled out the possibility of using non-parametric tests. The accessible studies were mostly based on reports of means and standard deviations. Student's t-test was therefore carried out to analyse the statistical significance of differences. A chi-square test for non-parametric data was used to determine the significance of differences in proportions.

The long term changes (T1 → T3) and short term changes (T1 → T2) were analysed using Wilcoxon's Signed-Rank test. To complete the description of this study and to enable comparison with other intervention studies Cohen's d [56] was also used, with the definitions small (0.20–0.49), moderate (0.50 – 0.79) and large effect size (≥ 0.80).

Since a relatively large number of statistical tests were performed, the possibility of the random significance of some results cannot be ruled out. A threshold p value of 0.01 was therefore deemed statistically significant.

## Results

### The families' problems at the outset of the treatment (T1)

The design of the study did not include a control group that could serve as a comparison at the outset. In order to give an idea of the occurrence and the extent of problems – whether they should be labelled "everyday problems" or could be considered to be of clinical significance – in the families participating in the study, the results have been compared to data from available community and clinical samples, preferably Swedish ones (Tables 5 and 6).

**Table 5:** Intervention mothers at the outset (T1) and comparative data

**Scale**	**Intervention mothers**			**Community samples **♀				**Clinical samples **♂			
	n	Mean	sd	n	Mean	sd	P	n	Mean	sd	p
SPSQ^a) ^(couples)	66	3.11	.58	1081	2.52	.56	<.001	75	2.81	.59	.003
*incompetence*	*66*	*3.11*	*.78*		*2.27*	*.68*	*<.001*		*2.57*	*.84*	<.*001*
*role*	*66*	*3.84*	*.84*		*3.42*	*.82*	*<.001*		*3.88*	*.75*	*.766*
*isolation*	*66*	*2.65*	*.84*		*2.05*	*.72*	*<.001*		*2.21*	*.82*	*.002*
*spouse*	*66*	*2.68*	*1.03*		*2.25*	*.94*	*<.001*		*2.29*	*1.07*	*.030*
*health*	*66*	*3.17*	*.83*		*2.61*	*.88*	*<.001*		*3.09*	*.88*	*.581*
SPSQ (single)	24	3.36	.56								
*incompetence*	*24*	*3.46*	*.60*								
*role*	*24*	*3.72*	*.98*								
*isolation*	*24*	*2.93*	*.92*								
*health*	*23*	*3.22*	*.69*								
RQ^b) ^(B)	92	3.95	1.88	211	5.02	1.50	<.001				
RQ (D)	91	3.74	2.33	209	2.40	1.70	<.001				
LoL^c) ^past	92	4.67	2.47					103	4.4	2.3	.430
L-o-L present	92	5.22	2.07	2032	7.30	1.48	<.001	103	5.2	2.0	.945
L-o-L future	89	7.92	1.81					102	7.7	2.0	.429
								Clinical sample ♀ & ♂			
ISSI^d) ^total	93	16.06	7.98					103	16.3	6.2	.813
*AVAT*	*92*	*4.64*	*1.72*					*103*	*4.4*	*1.6*	*.314*
*ADAT*	*92*	*5.08*	*3.20*					*103*	*5.9*	*2.9*	*.062*
*AVSI*	*93*	*2.04*	*1.80*					*103*	*2.0*	*1.7*	*.873*
*ADSI*	*93*	*4.37*	*2.77*					*103*	*4.4*	*2.5*	*.936*
SDQ^e) ^total	37	19.24	5.75	260	6.15	5.24	<.001	62	16.71	7.23	.073
SDQ impact	37	3.54	2.40		0.34	1.16	<.001		3.14	2.76	.466
*emotional*	*37*	*4.00*	*2.15*		*1.60*	*1.84*	*<.001*		*4.50*	*2.60*	*.327*
*conduct*	*37*	*4.86*	*2.00*		*1.09*	*1.29*	*<.001*		*3.23*	*2.23*	*<.001*
*hyperactivity*	*37*	*6.65*	*3.09*		*2.38*	*2.18*	*<.001*		*6.00*	*2.83*	*.288*
*peer*	*37*	*3.73*	*2.12*		*1.15*	*1.90*	*<.001*		*3.03*	*2.40*	*.146*
*prosocial*	*37*	*6.62*	*2.13*		*8.62*	*1.50*	*<.001*		*7.00*	*2.20*	*.402*

GHQ 12^f) ^*Prop. of poor psychol. health*	93	78.3%		8792	25.6%		*<.001*				

Student's t-test; statistical significance set at p < .01

^a) ^The Swedish Parenthood Stress Questionnaire: *Incompetence; Role restriction; Social isolation; Spouse relationship problems; Health problems*. Low values are desirable.

^b) ^The Relationship Questionnaire: B – High values are desirable; D – Low values are desirable.

^c) ^Cantril's Self-Anchoring Ladder of Life Satisfaction. High values are desirable.

^d) ^The Interview Schedule for Social Interaction;*(AVAT) Availability of attachment; (ADAT) Adequacy of attachment; (AVSI) Availability of social integration; (ADSI) Adequacy of social integration*. High values are desirable.

^e) ^The Strengths and Difficulties Questionnaire: *Emotional symptoms; Conduct problems; Hyperactivity; Peer problems; Prosocial behaviour*. Low values are desirable except for prosocial behaviour where high values are desirable.

^f) ^The General Health Questionnaire.

**Table 6:** Intervention fathers at the outset (T1) and comparative data

**Scale**	**Intervention Fathers**			**Community samples **♂				**Clinical samples **♂			
	n	Mean	Sd	n	Mean	sd	P	n	Mean	sd	p
SPSQ (couple)	51	2.72	.59					65	2.39	.50	.002
*incompetence*	*51*	*2.53*	*.72*						*2.02*	*.57*	<.001
*role*	*51*	*3.29*	*.81*						*3.23*	*.86*	*.703*
*isolation*	*51*	*2.61*	*.66*						*2.18*	*.73*	*.001*
*spouse*	*50*	*2.48*	*.81*						*1.98*	*.79*	*.001*
*health*	*51*	*2.70*	*.85*						*2.57*	*.81*	*.403*
SPSQ (single)	8	2.81	.78								
*incompetence*	*8*	*2.81*	*.87*								
*role*	*8*	*3.06*	*1.18*								
*isolation*	*8*	*2.85*	*.75*								
*health*	*8*	*2.31*	*.97*								
RQ (B)	60	4.13	1.71	192	4.88	1.48	.001				
RQ (D)	60	2.95	2.06	188	2.57	1.67	.149				
LoL past	59	5.20	2.23					47	5.2	2.1	1.000
L-o-L present	59	5.95	1.92					47	5.5	1.9	.231
L-o-L future	59	7.58	1.78					46	7.4	1.8	.610
								Clinical sample ♀ & ♂			
ISSI total	60	18.73	7.64					103	16.3	6.2	.028
*AVAT*	*60*	*4.73*	*1.53*	*83*	*5.1*	*1.4*	.*136*	*103*	*4.4*	*1.6*	*.199*
*ADAT*	*60*	*6.15*	*3.09*	*83*	*7.6*	*2.6*	*.003*	*103*	*5.9*	*2.9*	.*605*
*AVSI*	*60*	*2.58*	*1.86*	*83*	*3.0*	*1.7*	*.163*	*103*	*2.0*	*1.7*	*.044*
*ADSI*	*60*	*5.27*	*2.64*	*83*	*6.5*	*1.8*	*.199*	*103*	*4.4*	*2.5*	*.038*
SDQ total	25	17.92	6.47								
SDQ impact	25	2.76	2.89								
*emotional*	*25*	*3.56*	*2.36*								
*conduct*	*25*	*4.32*	*1.91*								
*hyperactivity*	*25*	*6.84*	*2.94*								
*peer*	*25*	*3.20*	*1.80*								
*prosocial*	*25*	*6.36*	*2.61*								

GHQ 12 *Prop. of poor psychol.. health*	60	43.3%		7126	18.6%		<.001				

Student's t-test; statistical significance set at p < .01

The mothers participating in the study showed a statistical significant higher degree of parental stress as measured by SPSQ compared to a community sample formed by 1500 randomly selected mothers with children aged from 6 months up to 3 years. Both fathers and mothers displayed significantly higher degrees of stress than a clinical sample consisting of 104 families seeking help for their children from a Specialist Child Health Centre [57]. The single parents showed even higher degrees of parental stress. The parents' attachment patterns differed from those of a community sample of 500 randomly selected families with children up to 6 years of age from the western region of Sweden [58]. The RQ results showed that the parents in this study had a significantly *lower *degree of secure attachment B than parents in the community sample and the mothers showed a *higher *degree of disorganized attachment D than mothers in the community sample.

The parents' mental health as measured with GHQ12 differed significantly (p < .001) from that of a sample of 7126 men and 8792 women aged 16 – 44 in an annual, national public health survey conducted by the Swedish National Institute of Public Health [59]. With a cut-off value of 3, 78.3% of the mothers and 43.3% of the fathers reported poor psychological health versus 25.6% for women and 18.6% for men in the community sample. There are no available data from Swedish community samples concerning the parents' present and expected life satisfaction as measured with Cantril's ladder. The instrument has, however, recently been used in a Dutch study [60] on a sample of 2032 mothers with children aged 1–3 years, recruited from community records of several cities and towns in the western region of the Netherlands. The mothers in our study made a significantly lower assessment of their current life satisfaction. The levels in our study are consistent with data from a Swedish study [61] comprising parents of children aged 3 – 9 who had been clinically assessed by professionals as displaying behaviour management problems.

Data from a Swedish sample concerning social support as measured with ISSI were based on 83 middle-aged men [51], and indicated on all four subscales a more favourable result than those of the fathers in our study, but only the differences in adequacy of attachment is statistically significant. In a recent Swedish study [52], data were presented from three psychiatric samples. The parents (results from both fathers and mothers) in our study are comparable with a sample consisting of patients aged 18 – 55 years (both men and women) from an outpatient unit for people with long-term mental illness, mainly psychosis.

The children's problems, as measured with the SDQ, deviated considerably from a Swedish community sample, consisting of the parents of 450 children, 5–14 years old, randomly selected from the population register [62]. The clinical comparison sample consists of children from four child psychiatric outpatient clinics in Sweden, with a mean age of 10 years. The children in our study displayed more severe problems in every subscale except emotional symptoms. The difference in conduct problems was statistically significant. The average scores were above cut-off scores for psychiatric cases [55] on the total score, the impact score and all of the sub-scales except for the prosocial scale where they were even.

At the outset of treatment (T1) the mothers showed a higher degree of problem load than the fathers on almost all scales. The only exceptions consisted of the mothers' somewhat more positive rating of the future than the fathers, and the fathers' higher rating of hyperactivity problems in the children and their lower rating of prosocial behaviour.

To sum up the results from commencement of treatment, the parents in this study had considerable problems in all areas examined. In the families where the children's problems have also been measured (children from the age of four) it appeared that the children undergoing treatment had problems of a nature and degree otherwise found in psychiatric populations.

### Long term changes (after 18 months (T3)) and short term changes (after 6 months (T2))

We found a clear general trend towards a positive development from T1 to T2 and this development was also reinforced from T2 to T3 (Tables 7 and 8). This trend was stronger for mothers (Additional file 1, Table S1) than for fathers (Additional file 1, Table S2). The gender differences will – for space reasons – be further analyzed and discussed in a forthcoming article.

**Table 7:** Parents' assessments at T1, T2 & T3

	**T1**			**T2**			**T3**		
**Scale**	*n*	Mean	*sd*	*n*	Mean	*sd*	*n*	Mean	*sd*
SPSQ (couples) total stress	117	2.94	.61	108	2.78	.55	103	2.67	.59
*incompetence*	*117*	*2.86*	*.81*	*108*	*2.70*	*.75*	*103*	*2.53*	*.74*
*role restriction*	*117*	*3.60*	*.87*	*108*	*3.37*	*.84*	*104*	*3.28*	*.89*
*isolation*	*117*	*2.63*	*.77*	*108*	*2.48*	*.74*	*103*	*2.38*	*.79*
*spouse*	*116*	*2.60*	*.94*	*109*	*2.52*	*.88*	*94*	*2.53*	*.95*
*health*	*117*	*2.97*	*.87*	*109*	*2.81*	*.81*	*103*	*2.64*	*.86*
SPSQ (single parents) total stress	32	3.22	.66	35	2.93	.68	32	2.75	.63
*incompetence*	*32*	*3.29*	*.72*	*35*	*2.99*	*.77*	*32*	*2.79*	*.74*
*role restriction*	*32*	*3.56*	*1.05*	*35*	*3.37*	*1.04*	*32*	*3.22*	*.97*
*isolation*	*32*	*2.91*	*.87*	*35*	*2.56*	*.88*	*32*	*2.33*	*.92*
*health*	*31*	*2.98*	*.86*	*35*	*2.68*	*.76*	*32*	*2.53*	*.90*
RQ (B)	152	4.02	1.81	143	4.20	1.78	135	4.50	1.67
RQ (D)	151	3.42	2.26	143	3.01	2.09	135	2.84	2.00
Cantril's L-o-L present	151	5.50	2.04	142	6.30	2.05	131	6.99	1.64
Cantril's L-o-L future	148	7.78	1.79	139	7.96	1.80	131	8.28	1.33
GHQ12	153	4.46	3.37	144	3.42	3.30	136	2.70	3.00
ISSI	153	17.11	7.93	143	17.99	7.25	136	19.36	7.03
*AVAT*	*152*	*4.68*	*1.64*	*143*	*4.72*	*1.56*	*135*	*5.16*	*1.28*
*ADAT*	*152*	*5.50*	*3.19*	*143*	*5.89*	*3.00*	*136*	*6.29*	*2.96*
*AVSI*	*153*	*2.25*	*1.84*	*143*	*2.22*	*1.79*	*136*	*2.44*	*1.83*
*ADSI*	*153*	*4.72*	*2.75*	*143*	*5.15*	*2.69*	*136*	*5.49*	*2.68*
SDQ total difficulties	62	18.71	6.03	59	15.92	6.74	56	14.21	7.37
SDQ impact	62	3.23	2.61	59	1.53	2.32	56	1.50	2.54
*emotional symptoms*	*62*	*3.82*	*2.23*	*59*	*3.47*	*2.48*	*56*	*2.71*	*2.10*
*conduct problems*	*62*	*4.65*	*1.97*	*59*	*3.78*	*2.04*	*56*	*3.36*	*2.34*
*hyperactivity*	*62*	*6.73*	*3.01*	*59*	*5.93*	*2.91*	*56*	*5.43*	*2.96*
*peer problems*	*62*	*3.52*	*2.00*	*59*	*2.73*	*2.26*	*56*	*2.71*	*2.08*
*prosocial behaviour*	*62*	*6.52*	*2.32*	*59*	*6.69*	*2.19*	*56*	*7.27*	*2.33*

**Table 8:** Parents' long term changes T1→T3 and short term changes T1→T2

	**Long term T1→T3**				**Short term T1→T2**			

**Scale**	d	Z	p		d	Z	p	
SPSQ (couples) total stress	.45	-4.539	<.001	***	.28	-3.643	<.001	***
*incompetence*	*.42*	*-4.678*	<.001	***	*.20*	*-2.812*	*.005*	**
*role restriction*	*.36*	*-3.964*	<.001	***	*.27*	*-2.809*	*.005*	**
*social isolation*	*.33*	*-2.678*	*.007*	**	*.20*	*-1.945*	*.052*	
*spouse relationship problems*	*.07*	*-.586*	*.558*		*.09*	*-.292*	*.770*	
*health problems*	*.38*	*-3.795*	<.001	***	*.19*	*-1.890*	*.059*	
SPSQ (single) total stress	.73	-3.375	.001	**	.43	-3.015	.003	**
*incompetence*	*.69*	*-3.084*	*.002*	**	*.41*	*-2.440*	*.015*	
*role restriction*	*.34*	*-2.469*	*.014*		*.18*	*-.931*	*.352*	
*social isolation*	*.65*	*-2.611*	*.009*	**	*.40*	*-2.973*	*.003*	**
*health problems*	*.51*	*-1.732*	*.083*		*.37*	*-1.948*	*.051*	
RQ (B)	.28	-2.851	.004		.10	-.972	.331	
RQ (D)	.27	-3.426	.001	**	.19	-2.202	.028	
Cantril's L-o-L present	.80	-6.335	<.001	***	.39	-4.093	<.001	***
Cantril's L-o-L future	.31	-3.090	.002	**	.10	-1.606	.108	
GHQ12	.55	-5.466	<.001	***	.31	-4.051	<.001	***
ISSI	.30	-2.636	.008	**	.12	-1.271	.204	
*AVAT*	*.33*	*-2.361*	*.018*		*.03*	*-.522*^a)^	*.602*	
*ADAT*	*.26*	*-2.187*	*.029*		*.14*	*-1.166*	*.244*	
*AVSI*	*.10*	*-1.018*	*.308*		*-.02*^a)^	*-.585*^a)^	*.559*	
*ADSI*	*.28*	*-3.400*	*.001*	**	*.16*	*-2.313*	*.021*	
SDQ total difficulties	.68	-4.254	<.001	***	.44	-4.167	<.001	***
SDQ impact	.67	-4.342	<.001	***	.69	-4.790	<.001	***
*emotional symptoms*	*.51*	*-2.764*	*.006*	**	*.15*	*-1.217*	*.224*	
*conduct problems*	*.60*	*-4.466*	<.*001*	***	*.43*	*-3.578*	<.*001*	***
*hyperactivity*	*.44*	*-3.199*	*.001*	**	*.27*	*-3.392*	*.001*	**
*peer problems*	*.40*	*-2.168*	*.030*		*.37*	*-2.920*	*.004*	**
*prosocial behaviour*	*.32*	*-2.718*	*.007*	**	*.08*	*-1.164*	*.244*	

**d **Cohen's d; effect size small 0.20 – 0.49, moderate 0.50 – 0.79, large ≥ 0.80.

**Z **Wilcoxon Signed Ranks test; **p < 0.01, ***p < 0.001. Statistical significance in this study set at *p *< 0.01.

^a) ^changes in an unfavourable direction

#### Reduced experience of parental stress

The experience of parental stress was reduced from T1 to T2, and the stress continued to diminish from T2 to T3. The change from T1 to T3 was statistically significant for spouses (p <.001) as well as for single parents (p = .001) and the effect size (Cohen's d) was moderate for spouses (*d *= 0.45) and moderate to large for single parents (*d *= 0.73).

#### Changes in parental attachment

The outcomes considered of special importance concerning the patterns of attachment were changes regarding pattern B (secure attachment), where an increase is desirable, and for pattern D (fearful or disorganized), where, instead, a decrease is desirable.

The parents showed a certain development towards the desirable pattern of attachment B from T1 to T2, and a stronger reinforcement from T2 to T3. The change from T1 to T3 was significant (p = .004), but the effect size according to Cohen's d was small (*d *= 0.28). The negative pattern of attachment D decreased from T1 to T2, a trend that also continued between T2 and T3, but the effect size was still small (*d *= 0.27).

#### Improved mental health

The parents' improved mental health expressed as an average value improved considerably from T1 to T2, as well as from T2 to T3. The change was highly significant statistically (p < .001) and the effect size was considered to be medium (d = 0.55). The proportion of persons with good mental health (cut off = 3) altered significantly (p < .001) from 35.3% at T1 to 52.1% at T2 and 61% at T3.

#### Improved present and expected life satisfaction

The parents' present life satisfaction was significantly improved from T1 to T3, (p < .001) and their expected life satisfaction also improved considerably (p = .002). The effect size was large concerning present life satisfaction (*d *= 0.80) and small (*d *= 0.31) with regard to the future.

#### More satisfactory social support

A certain short-term improvement took place from T1 to T2 and a more marked change was visible from T2 to T3. Wilcoxon's test showed a significant change from T1 to T3 (p = .008). The effect size was small as measured with Cohen's d (*d *= 0.30). On the sub-scales the effect size was next to non-existent (*d *= 0.10) for access to a social network, but significant and clear, albeit small, to adequacy of attachment.

#### Problem reduction with the children and reduced impact of the problems

The total symptom charge was significantly reduced from T1 to T3 (p < .001) and the effect size was of medium size (*d *= 0.68). The effect of the problems in the lives of the children and the families was also significantly reduced (p < .001), with a medium effect size (*d *= 0.67). The most important changes concerned conduct problems, which corresponds well with the problem description given by the parents at the outset.

When calculating the effect size concerning SDQ a measure called *added value *is sometimes used which takes into account a certain amount of "self-healing". In this study the measure of added value is 2.56, which would give an effect size of 0.51.

### Summary

The results of the study showed that the subjective assessment of parents partaking in parent-child interventions was that less parental stress was experienced after six months, with the exception of factors concerning the way in which the spouse relationship had been influenced. The parents' ways of relating to other people (patterns of attachment) had developed in a positive direction: their mental health had improved, as had their present and expected life satisfaction. The possibility of obtaining social support had increased – not primarily through a larger network but through experiencing the existing network as being more adequate. Finally the children's problems – especially conduct problems – had decreased, as had their effect in their daily life. The positive development in all these areas had continued and been reinforced eighteen months after the outset of the treatment. As can be seen from Tables 7 and 8, the variables under examination exhibited different patterns of improvement: there are "quick starters", which are more evident during the six first months (e.g. SDQ Impact); "slow starters" that improve over time (e.g. aspects of perceived social support) and others where the development seems to have taken a more even course (e.g. life satisfaction).

## Discussion

### Positive impact of a multi-modal approach to parent-child intervention

The main result of this study is that the families experienced a manifest improvement during the period of intervention. This improvement concerned all the aspects studied and led to an experience of increased mental wellbeing, increased faith in the future, reduced parental stress, greater possibilities of obtaining social support, positive changes in the way of relating to other people and a reduction of the impact of the problems pertaining to the children on everyday life. A clear pattern was visible: there was improvement after six months in all the areas studied and a continued and reinforced development was observed a year later.

With regard to the discussion of whether "less is more" or "more is better" the centres in this study endeavour to match the extent of the intervention to the needs of each family and there is a readiness to meet needs on different ecological levels and to choose different ports of entry in the interaction treatment. This approach supports the standpoint that "less is more" is relevant for some whereas "more is better" is more relevant for others [63]. There are families whose treatment may be restricted for instance to a limited number of Marte Meo-sessions with a narrow focus, but there are also families with a long history of mistrust of authorities to surmount before a collaborative relationship can be established and treatment can start. The differential patterns of improvement described above may reflect the variation of needs – the immediate impact of a child's behavioural problems can change rapidly whereas the parent's way of relating to other people seem to alter more slowly. At this stage the present study cannot claim to add much evidence on the question of "less" or/and "more". Further analysis of the dataset will, however, shed light on this issue with respect to the families in this study.

The tendencies were, with a few exceptions, similar for mothers and fathers but improvement was considerably stronger for mothers. The manifest and intriguing gender differences with regard to problem weight at the outset and improvement during the intervention will – as already noted – be further addressed in a forthcoming article.

One interesting result was that the level of dropout from treatment was low. There were only ten undesired or unplanned interruptions of the treatment, and when they occurred they were related to external circumstances. This result was unexpected since problems with high levels of dropout often are encountered in the literature concerning interventions in early childhood [64], and several studies have shown an attrition of 40–60% in children and families who began outpatient treatment services [65,66]. Attention has therefore been drawn to the need for interventions designed to improve commitment and decrease attrition [67], and Staudt [68] emphasizes, as did Cook [28], that research on interventions must include their acceptability to clients and their potential to reach and engage the families of at-risk children.

This raises questions about which aspects of the intervention in this study contributed to the low dropout levels. Successful negotiation and acceptance by the therapist and client of the goals, tasks and techniques have been found to increase engagement and hope [69]. In their research concerning barriers to treatment participation Kazdin and Wassell [70] point out the importance of the parents' perceived relevance of treatment. The principle adopted by the centres in this study that the goals and means of the intervention should be established through a dialogue between the parents and the therapist might therefore be a vital element. This is corroborated by a Swedish study [71] with 4–12-year-old children who displayed externalizing behaviour problems – using partly the same therapeutic approach – where there were no dropouts in the intervention group after the intervention had begun.

Another reason for the low number of dropouts from treatment might be that the intervention is adapted to the needs of each family. A mismatch – either way – between a family's assistance needs and the extent of the intervention can jeopardize the families' motivation to participate. The low number of dropouts from treatment has led to a very limited attrition from the study, which is a major strength since it implies that the results we have obtained have a high validity. As the study was a naturalistic one, it is the effectiveness of the centres' everyday practice that we are measuring. There is, therefore, no need to fear that the results depend upon special conditions during the intervention period. Another essential merit in this study lies in the fact that the change has been measured both in a short term and a long-term perspective. The long-term improvements in this study raise questions about what happens in an even longer perspective, especially since the results suggest that the notion of sleeper effects is of relevance in this kind of intervention programme.

### Variables of clinical importance

One of the most important reasons for seeking help was aggression in the child. This is of great interest as aggression and other anti-social behaviour – especially in children below 12 – is one of the main predictors for continued negative development [72]. Since a meta-analysis [73] has shown that aggressive behaviour tends to remain stable in all age groups when untreated, it is of utmost importance to provide effective treatment programmes for families.

Most of the results in this study, however, relate to improvements in the parents and an important question concerns which of these aspects may be considered important from a clinical perspective. A secure attachment is an important protective factor for a child growing up in a risk environment [74] and a disorganized attachment is a serious risk factor for externalizing problems [75]. Within attachment research, questions concerning the stability of patterns of attachment over time are studied and discussed as well as to what extent patterns of attachment are "inherited" by one generation from another. There is clear evidence of the importance of the parents' own attachment patterns for the child's possibility to develop a secure attachment [76]. This could imply that even small changes in a positive direction – an increased proportion of secure attachment and a reduced proportion of fearful/disorganized attachment – might be of significant importance for the children's development.

There is also strong evidence [13] indicating that the mother's mental health and well-being affect the child's development. Improved mental well-being should therefore be of vital importance. Likewise, the experience of parental stress is important. Anderson [77] has shown that the experience of stress is associated with a heightened risk of anxiety in the child, which indicates that stress reduction is clinically relevant.

In a study [78] comprising 152 infant parents there was an association between social support and increasingly positive parent-child activities over time, but this effect was mediated by mothers' attachment styles. It is considered important to reduce the feelings of relationship anxiety, and the authors consider that parenting interventions can achieve this by actively building on parents' successful social experiences within the framework of the intervention. This concurs with the emphasis of the centres on the therapist-parent relationship [79].

### Limitations

A limitation of this present study is its lack of a control group. For ethical and practical reasons it was not possible to create one and we cannot therefore say with certainty what the development would have been like for these families had they not received help. A crucial question is whether results corresponding to those displayed by the families in this study could be obtained through spontaneous improvement. In a prospective study [80] 2587 children were followed up 3 years after the original survey for a sub-sample of the 1999 British Child and Adolescent Mental Health Survey. Latent mental health scores (i.e. combined information from multiple informants) showed strong stability over time (r = 0.71). A poorer outcome was associated for instance with externalizing as opposed to emotional symptoms and after exposure to parental mental illness. The authors conclude that there is a need for effective intervention with children with impairing psychopathology, since they are unlikely to improve spontaneously. The predictors of change in mental health were closely comparable across the range of initial SDQ scores, suggesting that they operated in a similar manner regardless of the initial level of (mal)adjustment.

A control group of "community families" would have enabled better comparison with respect to the burden of problems at the outset of treatment. Though the comparative data presented do not offer a perfect match – for more detailed information about the samples see references – they do contribute to the description of the subjects in the study.

Zaslow et al [81] have shown that self-reports have a predictive value and that they are an appropriate choice when budgets or time are limited. It was logical to prioritize the parents' subjective perspective in this study, but we realize that deeper knowledge could be attained if supplemented by observations/assessments; e.g. parent-child interaction, the children's attachment, health data and how the children function in day care.

## Conclusions and directions for future research

This study has shown that it is possible to reach mothers and fathers with parenting problems, and to create an intervention program with very low dropout levels. This is of special importance since aggressive behaviour by the children was one of the most important reasons for seeking help in this study. Aggression in childhood has been shown to be a serious risk factor for further negative development, and families facing these problems have often displayed high levels of dropout.

The role of fathers in parent-child interaction interventions remains unexplored. Future research regarding fathers in parent-child interventions is of special importance so that the continued development of these interventions will be tailored to the needs of the fathers as well as these of the mothers. Clinicians also need more empirical knowledge on the question of "less" or/and "more", and this will be the focus of another forthcoming article from this study.

Another important and neglected aspect is the children's own experiences of participation in parent-child interventions. We have addressed parents' subjective accounts of participating in treatment at the four centres in a previous study [79], but this perspective should be complemented by assessments of the parents, the children and the interaction from a third-person perspective.

Since living conditions in different cultures may create different problems there is a demand for further knowledge about parent-child interventions with various designs from various cultural contexts. There is therefore a need to deepen our understanding of what support society should offer to vulnerable fathers and mothers in order to help them to provide "a secure base" for their children.

## Competing interests

The authors declare that they have no competing interests.

## Authors' contributions

KN conceived the study, shared responsibility for the design and was responsible for the data collection, performed the statistical analysis and drafted the manuscript. IE shared responsibility for the design and helped to draft the manuscript. Both authors read and approved the final manuscript.

## Supplementary Material

Additional file 1 **Table S1 and Table S2.** Mothers' long term changes T1 → T3 and short term changes T1 → T2, Fathers' long term changes T1 → T3 and short term changes T1 → T2. Click here for file
