# Supplementary material for: Parents' assessment of parent-child interaction interventions – a longitudinal study in 101 families
Source: Child Adolesc Psychiatry Ment Health. 2009 Mar 10;3:8. doi: 10.1186/1753-2000-3-8 (PMC2669452; doi:10.1186/1753-2000-3-8)
Supplement: Additional file 1 — Table S1 and Table S2. Mothers' long term changes T1 → T3 and short term changes T1 → T2, Fathers' long term changes T1 → T3 and short term changes T1 → T2. [file 1753-2000-3-8-S1.doc]

## Table S1 - Mothers’ long term changes T1 → T3 and short term changes T1 →T2

|  | | **T1** | | | **T2** | | | **T3** | | | Long term T1 → T3 | | | Short term T1 →T2 | | |
| --- | --- | --- | --- | --- | --- | --- | --- | --- | --- | --- | --- | --- | --- | --- | --- | --- |
| **Scale** | | n | Mean | sd | n | Mean | sd | n | Mean | sd | d | Z | p | d | Z | p |
| SPSQ (couples) | | 66 | 3.11 | .58 | 62 | 2.95 | .54 | 58 | 2.78 | .61 | .56 | -3.759 | <.001 | .29 | -3.314 | .001 |
|  | *incompetence* | *66* | *3.11* | *.78* | *62* | *2.97* | *.71* | *58* | *2.71* | *.76* | *.53* | *-3.810* | <.001 | *.17* | *-1.972* | *.049* |
|  | *role restriction* | *66* | *3.84* | *.84* | *62* | *3.55* | *.86* | *59* | *3.45* | *.86* | *.46* | *-3.480* | *.001* | *.34* | *-3.023* | *.003* |
|  | *isolation* | *66* | *2.65* | *.84* | *62* | *2.50* | *.78* | *58* | *2.33* | *.88* | *.37* | *-1.940* | *.052* | *.18* | *-1.631* | *.103* |
|  | *spouse* | *66* | *2.68* | *1.03* | *63* | *2.64* | *.99* | *52* | *2.55* | *.99* | *.13* | *-.784* | *.433* | *.04* | *-.037^a)^* | *.971* |
|  | *health* | *66* | *3.17* | *.83* | *63* | *3.00* | *.81* | *58* | *2.85* | *.92* | *.37* | *-2.723* | *.006* | *.21* | *-1.749* | *.080* |
| SPSQ (singel) | | 24 | 3.36 | .56 | 26 | 3.00 | .67 | 24 | 2.71 | .61 | 1.14 | -3.654 | <.001 | .59 | -2.825 | .005 |
|  | *incompetence* | *24* | *3.46* | *.60* | *26* | *3.00* | *.75* | *24* | *2.77* | *.70* | *1.06* | *-3.509* | <.001 | *.68* | *-3.104* | *.002* |
|  | *role restriction* | *24* | *3.72* | *.98* | *26* | *3.54* | *.98* | *24* | *3.33* | *.92* | *.41* | *-2.257* | *.024* | *.18* | *-.263* | *.793* |
|  | *isolation* | *24* | *2.93* | *.92* | *26* | *2.60* | *.89* | *24* | *2.08* | *.86* | *.96* | *-3.255* | *.001* | *.36* | *-2.471* | *.013* |
|  | *health* | *23* | *3.22* | *.69* | *26* | *2.79* | *.76* | *24* | *2.52* | *.87* | *.90* | *-2.550* | *.011* | *.59* | *-2.065* | *.039* |
| RQ (B) | | 92 | 3.95 | 1.88 | 89 | 4.18 | 1.90 | 82 | 4.50 | 1.70 | .30 | -2.387 | .017 | .12 | -1.048 | .294 |
| RQ (D) | | 91 | 3.74 | 2.33 | 89 | 3.33 | 2.26 | 82 | 3.11 | 2.14 | .26 | -2.714 | .007 | .18 | -1.395 | .163 |
| L-o-L present | | 92 | 5.22 | 2.07 | 87 | 6.24 | 2.05 | 81 | 7.24 | 1.51 | 1.12 | -6.476 | <.001 | .50 | -4.043 | <.001 |
| L-o-L future | | 89 | 7.92 | 1.81 | 84 | 8.07 | 1.77 | 81 | 8.49 | 1.26 | .36 | -3.153 | .002 | .08 | -1.408 | .159 |
| GHQ12 | | 93 | 5.34 | 3.22 | 89 | 3.92 | 3.13 | 83 | 2.95 | 2.97 | .77 | -5.663 | <.001 | .45 | -4.422 | <.001 |
| ISSI | | 93 | 16.06 | 7.98 | 88 | 17.10 | 7.23 | 83 | 18.77 | 7.38 | .35 | -2.210 | .027 | .14 | -1.345 | .179 |
|  | *AVAT* | *92* | *4.64* | *1.72* | *88* | *4.74* | *1.62* | *83* | *5.16* | *1.40* | *.33* | *-1.987* | *.047* | *.06* | *-.324* | *.746* |
|  | *ADAT* | *92* | *5.08* | *3.20* | *88* | *5.65* | *3.03* | *83* | *6.00* | *3.06* | *.29* | *-2.084* | *.037* | *.18* | *-1.812* | *.070* |
|  | *AVSI* | *93* | *2.04* | *1.80* | *88* | *1.94* | *1.70* | *83* | *2.31* | *1.82* | *.15* | *-.827* | *.409* | *-.06^a)^* | *-.844^a)^* | *.399* |
|  | *ADSI* | *93* | *4.37* | *2.77* | *88* | *4.77* | *2.69* | *83* | *5.30* | *2.77* | *.34* | *-2.838* | .005 | *.15* | *-1.626* | *.104* |
| SDQ total difficulties | | 37 | 19.24 | 5.75 | 35 | 16.43 | 6.97 | 34 | 14.38 | 8.07 | .71 | -3.317 | .001 | .44 | -3.010 | .003 |
| SDQ impact | | 37 | 3.54 | 2.40 | 35 | 1.74 | 2.65 | 34 | 1.71 | 2.74 | .71 | -3.441 | .001 | .71 | *-3.589* | <.001 |
|  | *emotional* | *37* | *4.00* | *2.15* | *35* | *3.86* | *2.67* | *34* | *2.62* | *2.24* | *.63* | *-2.646* | *.008* | *.06* | *-.476* | *.634* |
|  | *conduct* | *37* | *4.86* | *2.00* | *35* | *3.66* | *2.24* | *34* | *3.47* | *2.61* | *.61* | *-3.458* | *.001* | *.57* | *-3.471* | *.001* |
|  | *hyperactivity* | *37* | *6.65* | *3.09* | *35* | *6.06* | *2.94* | *34* | *5.47* | *3.09* | *.38* | *-2.332* | *.020* | *.20* | *-1.898* | *.058* |
|  | *peer* | *37* | *3.73* | *2.12* | *35* | *2.86* | *2.18* | *34* | *2.82* | *2.30* | *.41* | *-1.836* | *.066* | *.40* | *-2.028* | *.043* |
|  | *prosocial* | *37* | *6.62* | *2.13* | *35* | *6.63* | *2.29* | *34* | *7.44* | *2.25* | *.37* | *-2.414* | *.016* | *.00* | *-.816* | *.415* |
| *d* = effect size (Cohen) small 0.20– .49, moderate 0.50– .79, large ≥0.80. Z = Wilcoxon Signed Ranks test . ^a)^ changes in an unfavourable direction | | | | | | | | | | | | | | | | |

## Table S2 - Fathers’ long term changes T1 → T3 and short term changes T1 →T2

|  | | **T1** | | | **T2** | | | **T3** | | | Long term T1→T3 | | | Short term T1 →T2 | | |
| --- | --- | --- | --- | --- | --- | --- | --- | --- | --- | --- | --- | --- | --- | --- | --- | --- |
| **Scale** | | n | Mean | sd | n | Mean | sd | n | Mean | sd | d | Z | p | d | Z | p |
| SPSQ (couples) | | 51 | 2.72 | .59 | 46 | 2.55 | .48 | 45 | 2.52 | .52 | .35 | -2.561 | .010 | .31 | -1.593 | .111 |
|  | *incompetence* | *51* | *2.53* | *.72* | *46* | *2.34* | *.65* | *45* | *2.30* | *.64* | *.34* | *-2.638* | *.008* | *.28* | *-2.008* | *.045* |
|  | *role restriction* | *51* | *3.29* | *.81* | *46* | *3.11* | *.75* | *45* | *3.05* | *.89* | *.28* | *-2.074* | *.038* | *.23* | *-.605* | *.545* |
|  | *isolation* | *51* | *2.61* | *.66* | *46* | *2.45* | *.69* | *45* | *2.42* | *.66* | *.29* | *-1.836* | *.066* | *.24* | *-1.010* | *.313* |
|  | *spouse* | *50* | *2.48* | *.81* | *46* | *2.36* | *.66* | *42* | *2.52* | *.91* | *-.05^a)^* | *-.049^a)^* | *.961* | *.16* | *-.547* | *.584* |
|  | *health* | *51* | *2.70* | *.85* | *46* | *2.54* | *.73* | *45* | *2.36* | *.70* | *.44* | *-2.666* | *.008* | *.20* | *-.877* | *.381* |
| SPSQ (singel) | | 8 | 2.81 | .78 | 9 | 2.73 | .74 | 8 | 2.87 | .72 | -.08^a)^ | -.681^a)^ | .496 | .11 | -1.193 | .233 |
|  | *incompetence* | *8* | *2.81* | *.87* | *9* | *2.96* | *.87* | *8* | *2.85* | *.92* | *-.04^a)^* | *-.339^a)^* | *.734* | *-.17^a)^* | *-.914^a)^* | *.361* |
|  | *role restriction* | *8* | *3.06* | *1.18* | *9* | *2.87* | *1.10* | *8* | *2.90* | *1.12* | *.11* | *-.853* | *.394* | *.13* | *-1.363* | *.173* |
|  | *isolation* | *8* | *2.85* | *.75* | *9* | *2.43* | *.88* | *8* | *3.08* | *.68* | *-.32^a)^* | *-1.020^a)^* | *.308* | *.51* | *-1.590* | *.112* |
|  | *health* | *8* | *2.31* | *.97* | *9* | *2.36* | *.73* | *8* | *2.56* | *1.05* | *-.25^a)^* | *-1.194^a)^* | *.233* | *-.06^a)^* | *.000* | *1.000* |
| RQ (B) | | 60 | 4.13 | 1.71 | 54 | 4.22 | 1.57 | 53 | 4.51 | 1.56 | .23 | -1.589 | .112 | .05 | -1.589 | .112 |
| RQ (D) | | 60 | 2.95 | 2.06 | 54 | 2.5 | 1.68 | 53 | 2.42 | 1.70 | .28 | -2.076 | .038 | .24 | -1.844 | .065 |
| L-o-L present | | 59 | 5.95 | 1.92 | 55 | 6.38 | 2.06 | 50 | 6.57 | 1.77 | .33 | -1.346 | .178 | .22 | -1.221 | .222 |
| L-o-L future | | 59 | 7.58 | 1.78 | 55 | 7.81 | 1.86 | 50 | 7.93 | 1.39 | .22 | -.961 | .337 | .13 | -.842 | .400 |
| GHQ12 | | 60 | 3.10 | 3.16 | 55 | 2.60 | 3.42 | 53 | 2.30 | 3.04 | .25 | -.719 | .472 | .15 | -.646 | .518 |
| ISSI | | 60 | 18.73 | 7.64 | 55 | 19.40 | 7.12 | 53 | 20.28 | 6.40 | .22 | -1.399 | .162 | .09 | -.292 | .771 |
|  | *AVAT* | *60* | *4.73* | *1.53* | *55* | *4.69* | *1.49* | *52* | *5.15* | *1.07* | *.32* | *-1.290* | *.197* | *.-03^a)^* | *-1.396^a)^* | *.163* |
|  | *ADAT* | *60* | *6.15* | *3.09* | *55* | *6.27* | *2.93* | *53* | *6.75* | *2.77* | *.20* | *-.798* | *.425* | *.04* | *-.483^a)^* | *.629* |
|  | *AVSI* | *60* | *2.58* | *1.86* | *55* | *2.67* | *1.84* | *53* | *2.64* | *1.85* | *.03* | *-.651* | *.515* | *.05* | *-.166* | *.868* |
|  | *ADSI* | *60* | *5.27* | *2.64* | *55* | *5.76* | *2.60* | *53* | *5.78* | *2.54* | *.19* | *-1.788* | *.074* | *.19* | *-1.714* | *.086* |
| SDQ total difficulties | | 25 | 17.92 | 6.47 | 24 | 15.17 | 6.46 | 22 | 13.95 | 6.33 | .62 | -2.690 | .007 | .43 | -2.946 | .003 |
| SDQ impact | | 25 | 2.76 | 2.89 | 24 | 1.21 | 1.74 | 22 | 1.18 | 2.24 | .61 | -2.609 | .009 | .67 | -3.084 | .002 |
|  | *emotional* | *25* | *3.56* | *2.36* | *24* | *2.92* | *2.10* | *22* | *2.86* | *1.88* | *.33* | *-.950* | *.342* | *.29* | *-1.335* | *.182* |
|  | *conduct* | *25* | *4.32* | *1.91* | *24* | *3.96* | *1.76* | *22* | *3.18* | *1.89* | *.60* | *-2.681* | *.007* | *.20* | *-.984* | *.325* |
|  | *hyperactivity* | *25* | *6.84* | *2.94* | *24* | *5.75* | *2.92* | *22* | *5.36* | *2.82* | *.51* | *-2.190* | *.029* | *.37* | *-2.990* | *.003* |
|  | *peer problems* | *25* | *3.20* | *1.80* | *24* | *2.54* | *2.40* | *22* | *2.55* | *1.71* | *.37* | *-1.119* | *.263* | *.32* | *-2.341* | *.019* |
|  | *prosocial* | *25* | *6.36* | *2.61* | *24* | *6.79* | *2.08* | *22* | *7.00* | *2.49* | *.25* | *-1.373* | *.170* | *.18* | *-.854* | *.393* |
| *d* = effect size (Cohen) small 0.20 – 0.49, moderate 0.50 – 0.79, large ≥ 0.80. Z = Wilcoxon Signed Ranks test. ^a)^ changes in an unfavourable direction | | | | | | | | | | | | | | | | |
